# Norovirus Immunity and the Great Escape

**DOI:** 10.1371/journal.ppat.1002921

**Published:** 2012-10-18

**Authors:** Kari Debbink, Lisa C. Lindesmith, Eric F. Donaldson, Ralph S. Baric

**Affiliations:** 1 Department of Microbiology and Immunology, School of Medicine, University of North Carolina at Chapel Hill, Chapel Hill, North Carolina, United States of America; 2 Department of Epidemiology, Gillings School of Global Public Health, University of North Carolina at Chapel Hill, Chapel Hill, North Carolina, United States of America; University of Florida, United States of America

## Noroviruses Represent a Significant Worldwide Disease Burden

Noroviruses (NoVs), members of the Calicivirus family, are small, positive-polarity RNA viruses and the most important cause of human foodborne viral gastroenteritis worldwide. These viruses cause gastrointestinal disease, resulting in recurrent bouts of vomiting and diarrhea that typically last 24–48 hours. NoVs are transmitted via the fecal–oral route, most commonly through infected food or water or person-to-person contact, and result in 267 million infections [1] and over 200,000 deaths each year, mostly in infants and the elderly [2]. Vaccines and therapeutics are under development but face considerable challenges as there is no cell-culture system or small-animal model for human disease, and these viruses are highly heterogeneous and undergo antigenic variation in response to human herd immunity, further complicating our understanding of the complex immune interactions that regulate susceptibility and disease.

Despite these limitations, considerable progress has been made in understanding NoV adaptive immunity. This article discusses our current understanding of virus–host immune interactions that regulate host susceptibility, virus evolution, and protective immunity. We focus on virion structure, serologic relationships among strains, molecular mechanisms governing the changing antigenic landscape of human NoVs over time, cellular immunity, and relationships between human herd immunity, antigenic variation, and histoblood group antigen (HBGA) recognition, which are predicted to drive the emergence of new outbreak strains that target different human populations and/or afford escape from protective herd immunity. We discuss the implications of these observations on future vaccine design.

## Specific Host and Virus Genetic Factors Influence NoV Susceptibility, Evolution, and Immunity

**Figure 1:**
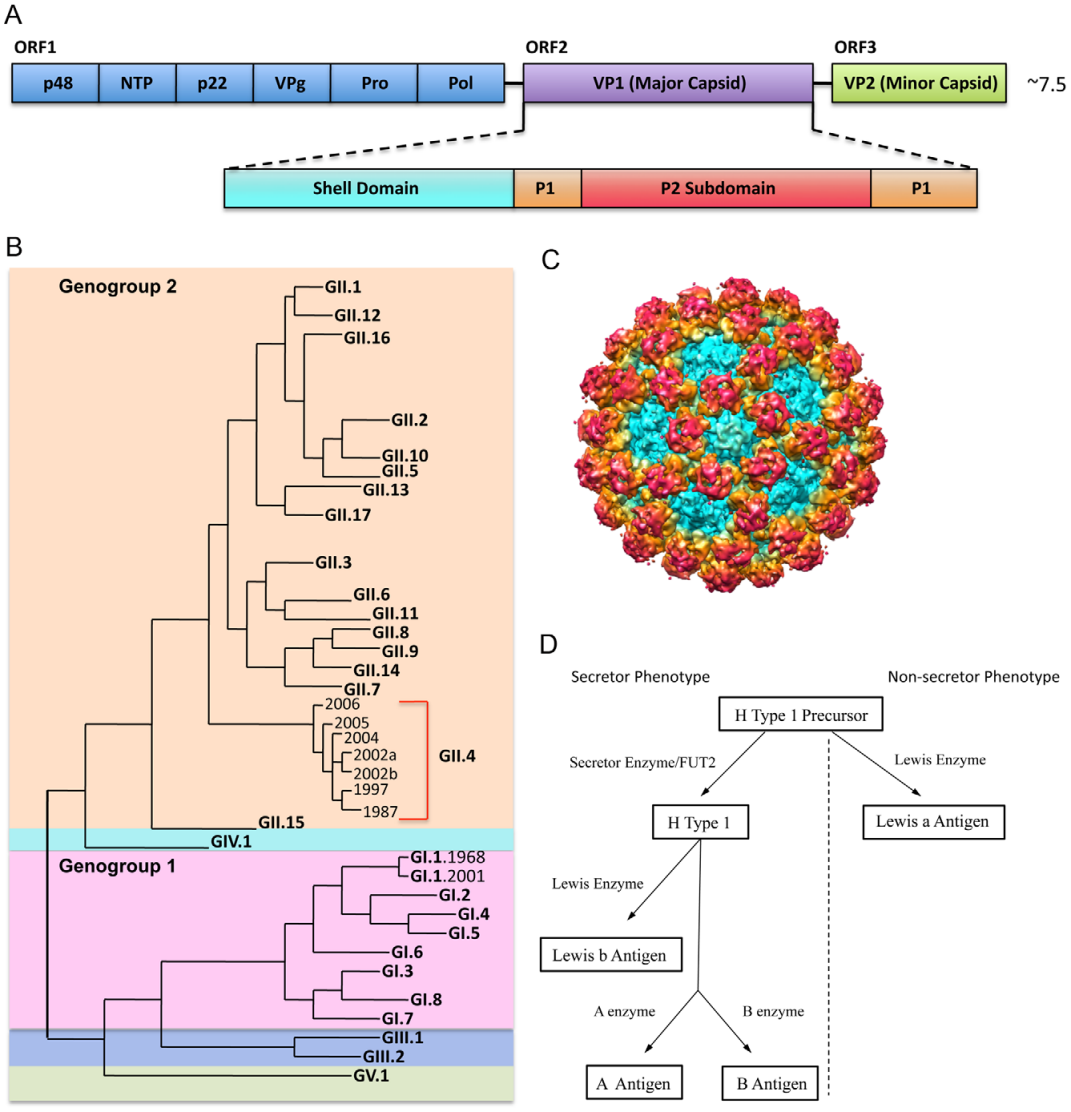
NoV Genetic Diversity, Structure, and Binding Ligand. **1A:** **NoV genome schematic.** The NoV genome encodes three open reading frames. ORF 1 encodes the nonstructural proteins (blue); ORF 2 encodes VP1, the major capsid protein (purple); and ORF 3 encodes VP2, the minor capsid protein (green). VP1 is further divided into the shell, which forms the base of the virion (teal); the P1 subdomain, which forms a stalk-like projection from the surface (orange); and the P2 subdomain, which is the most variable and surface-exposed area of the virion, contains ligand binding sites, and interacts with potentially neutralizing antibodies (red). **1B: NoV phylogenetic tree.** NoVs are divided into five genogroups. Genogroups 1 (pink) and 2 (orange) cause the majority of human disease. Genogroups are further divided into genotypes. Genotype GII.4 NoVs (red bracket) account for ∼80% of outbreaks. Genotype GI.1 NoVs are the prototypic Norwalk viruses. **1C: NoV capsid protein (VP1) cryo EM image.** Colors correspond approximately to the shell (teal), the P1 subdomain (yellow/orange) and the P2 subdomain (red). **1D. Secretor/non-secretor phenotype pathways.** Enzymes (Secretor or Lewis) add specific modifications to a precursor molecule. Individuals without a functional FUT2 gene cannot express HBGAs from the left branch of the pathway (left of the dotted line) on mucosal surfaces. For those without a functional FUT2 gene (non-secretors), the precursor molecule can still be modified by the Lewis enzyme to make Lewis a antigen (branch on the right side of the dotted line).

NoVs are divided into five genogroups (GI-GV), which differ by >60% based on capsid sequence [3], and GI and GII NoVs cause the majority of human disease (Figure 1A–C). Genogroups are further divided into genotypes, which differ by about 40%, with GI.1 as the prototypic “Norwalk” genotype and the GII.4 NoVs as the genotype responsible for the majority (80%) of outbreaks [4]. GII.4 NoVs in particular appear to accommodate a high level of sequence diversity and undergo positive selection in key surface-exposed residues, likely allowing for escape from herd immunity [1], [5]. Differences in evolution rates among different GI and GII NoVs have been attributed to receptor switching and effective population size, VP1 sequence space and structural plasticity, duration of herd immunity, and replication fidelity [1], [6]–[9].

HBGAs are a diverse family of carbohydrates expressed on mucosal surfaces where they serve as binding ligands and putative receptors for NoV. HBGAs are differentially expressed in individuals and binding to specific HBGAs varies by NoV strain. Expression of most HBGAs on mucosal tissues is dependent on the presence of a functional FUT2 gene, which codes for a fucosyltransferase that adds side chains to a precursor molecule. About 20% of people do not encode a functional FUT2 gene and are considered “non-secretors” (Figure 1D). Non-secretors are resistant to GI.1 (Norwalk virus) infection [10]; however, some other NoV strains are known to infect non-secretors, probably by attachment to Lewis carbohydrates [11]–[14]. GII.4 strains may predominate because the epidemic strains bind A, B, and O secretors, representative of 80% of the population. Antibodies that block virus binding to HBGA are considered “blockade antibodies” and are predicted to be neutralizing. Importantly, high prechallenge blockade antibody titers correlate with protection from infection following primary challenge and vaccination [15]. The development of more human challenge strains and therapeutic antibodies will be key for illuminating the complex relationships among HBGA affinity, host susceptibility, short and long-term immunity outcomes, and the mechanism of action by which blockade antibodies prevent infection.

## NoV Immunity: Humoral Immune Response

A handful of human challenge studies provide insight into the potential for protective immunity to NoVs. Short-term immunity has previously been established for GI.1 viruses [16], and a recent vaccine study found that intranasal vaccination with GI.1 VLPs protected against disease three weeks post vaccination [17]. The existence of long-term immunity is more controversial; however, multiple studies found protective responses against GI.1 were present six months after challenge in some but not all individuals [15], [17], [18]. Mucosal IgA responses to Norwalk virus indicate that an early salivary IgA response (days 1–5), rather than a late response, correlated with protection from infection in secretor-positive individuals; this suggests that previous strain exposure elicited a protective memory response against the challenge strain [10]. The rapid epidemic GII.4 strain replacement by new isolates every 3–7 years is consistent with protective, long-term herd immunity in a substantial portion of the population [19].

GI and GII antibodies are high in acute sera, while cross-blockade patterns are genogroup-specific [15], [20]. Sera against GI outbreaks are cross-blocking within the genogroup and are sometimes higher for a heterologous strain after infection [6], [20]; however, the blocking response does not extend to GII NoVs [21]. In contrast, sera against GII outbreaks have much higher strain-specific homologous responses and not broad GII blocking responses [22], [23]. These studies are complicated by complex preexposure histories in human populations, coupled with a very poor understanding of the serologic relationships among strains. Using mouse sera targeting single strains, reactivity across GI genotypes is about 5–10% of the homotypic response and is less than 5% between genogroups [24]. Simultaneous exposure of rodents or rabbits to multiple strains significantly boosts cross-reactive antibody responses, suggesting that complex patterns of cross reactivity may exist within multiple GI or multiple GII genotypes or that repeat and/or multivalent exposure selects for high-affinity antibodies that tolerate variation within target epitopes [24], [25].

**Figure 2:**
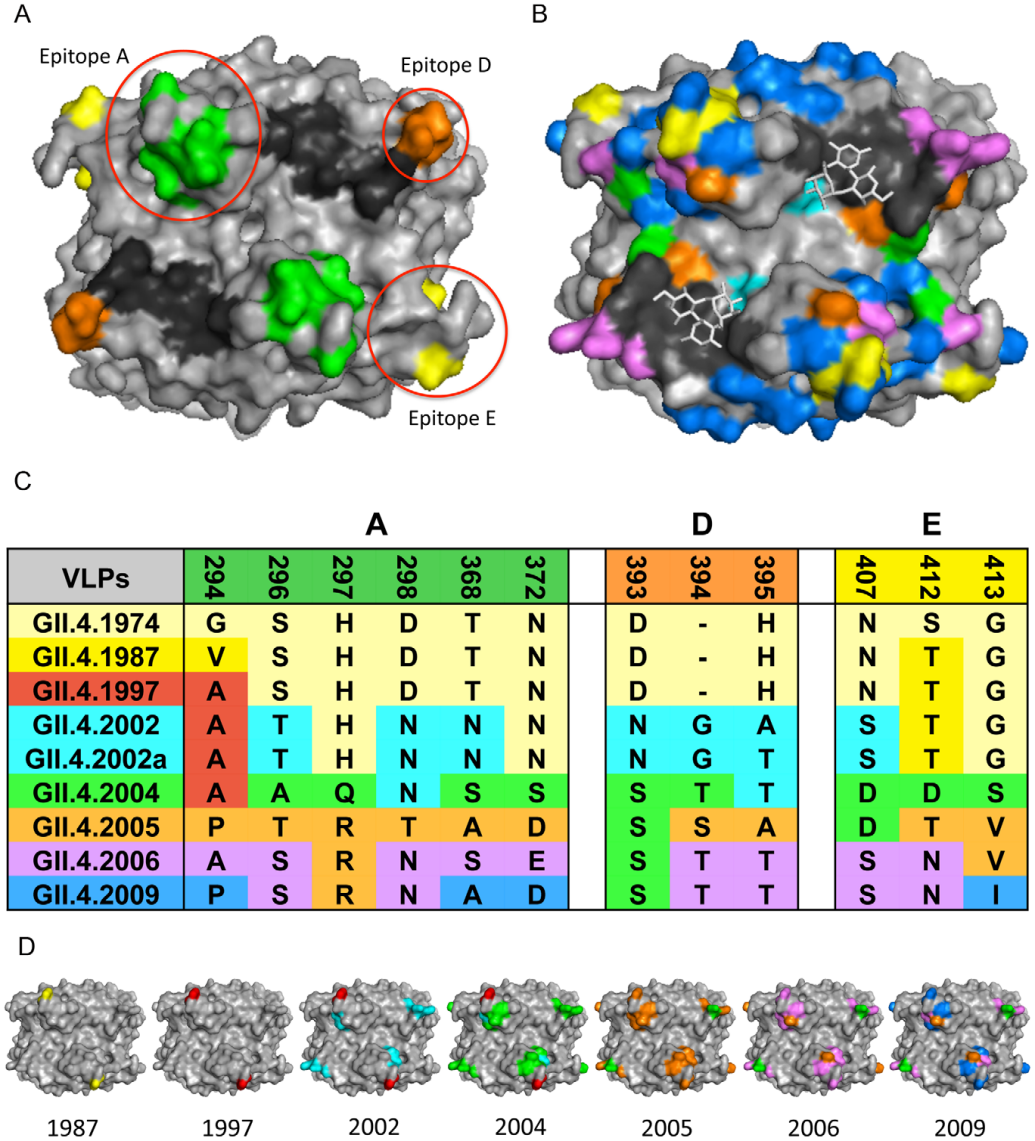
GII.4 NoV Variation over Time. **2A: GII.4 blockade epitopes.** Three blockade epitopes have been identified in GII.4 NoVs. Epitope A (residues 294, 296–298, 368, and 372; green), Epitope D (residues 393–395; orange), and Epitope E (residues 407, 412–413; yellow) all map to the P2 subdomain on the surface of the virion. The HBGA interaction sites are shown in black. **2B: GII.4 P2 subdomain variation over time.** Colored residues indicate change over time since 1974; changes present in 1987 = yellow, 1997 = red, 2002 = teal, 2004 = green, 2005 = orange, 2006 = purple, 2009 = blue, HBGA interaction sites = black, carbohydrates = white sticks. **2C: GII.4 NoV variation over time in blockade-epitope regions.** GII.4 NoV blockade epitopes undergo change over time, likely in response to human herd immunity. Colors indicate in which outbreak strain a particular residue change originated. **2D: Mapping of GII.4 variation over time in blockade-epitope regions.** Each VLP shows areas within blockade epitopes that change over time. Yellow indicates differences from 1974 present in 1987, 1997 = red, 2002 = teal, 2004 = green, 2005 = orange, 2006 = purple, and 2009 = blue. These blockade epitopes have continued to evolve in new outbreak strains since 2009.

Classic approaches for mapping epitopes cannot be applied to NoV because of the lack of a cell culture system for isolating antibody escape mutants. For G1 NoVs, point and deletion mutations have identified regions of VP1 targeted by antibodies [26], [27]. For GII.4 strains, epitope mapping has been done primarily by using bioinformatics approaches to identify rapidly evolving amino acid residues and exchanging these regions between strains [7]–[9], [28] (Figure 2B). This has allowed for precise mapping of key residues that drive antigenic change in response to human and rodent antibody binding and blockade responses. These mapping studies define key sites of antigenic change; however, the actual antibody binding epitope is usually conformational and likely includes proximal conserved and varying residues that contribute to escape from human herd immunity. A recent crystallography study mapped the binding of a cross-reactive GII monoclonal antibody in complex with a GII.10 P particle to a highly conserved, occluded site within the P1 subdomain, suggesting that the NoV P domain may accommodate high conformational flexibility [29].

Monoclonal antibodies that target distinct GII.4 strains demonstrate that antigenic variation is high and these strains are evolving in response to human herd immunity. These data also support the hypothesis that many human and mouse blockade monoclonal antibodies appear to target similar varying epitopes in GII.4 VLPs. Three blockade epitopes have been confirmed, designated A, D, and E [5], [7]–[9] (Figure 2A, C, D). Epitope A is substantially recognized by human polyclonal sera; Epitope D (residues 393–395) is especially interesting because this region also alters HBGA binding affinity [6], [8], [14]. Importantly, New Orleans 2009 and its recent derivatives demonstrate continued evolution in the major blockade epitopes, suggesting escape from GII.4–2006 herd immunity. These data support the hypothesis that antigenic changes that result in escape from herd immunity may also drive changes in HBGA affinities, altering population susceptibility patterns. While multiple blockade epitopes change over time, conserved, unmapped GII.4 blockade epitopes also exist [5]. While evidence for cross-blockade GII epitopes is limited [21], cross-blockade epitopes may be more common for GI strains, explaining the reduced frequency of disease patterns seen in human populations [20]. No GI or cross-GI and GII antibody blockade epitopes have been mapped, signaling an important priority for future studies. To further characterize the complexity of the molecular mechanisms driving antigenic variation, additional crystal structures in complex with strain, genotype, and genogroup-specific antibodies are needed to define complete epitopes, tease apart overlapping epitopes, and map the exact residues comprising important cross-reactive and cross-blockade epitopes.

## NoV Immunity: Cellular Immune Response

The role that T cells play in controlling NoV infection is complex and not well characterized. Human NoV infection or vaccination elicits a primarily CD4+ Th1 response, leading to increased secretion of IFN-gamma and IL-2 [13], [20]. One study using human-derived PBMCs found that T cell responses were more cross-reactive between GII strains with higher antigenic relatedness [13], while another study found that T cell responses toward alternate GI strains were more robust than the immunizing GI strain in some individuals [20]. Additional studies using a wider array of genotypes are needed to further characterize T cell responses and their relationships in controlling human infection.

## Important Considerations for NoV Therapeutic Design

NoVs are the primary cause of acute gastroenteritis and are responsible for hundreds of thousands of deaths worldwide, mostly in infants in the developing world. In developed countries, the elderly are particularly vulnerable to life-threatening infections [6]. Although few people die from NoV in developed countries, economic costs of NoV outbreaks are high due to lost productivity, lost wages, and decontamination costs; a single outbreak in a hospital setting can cost upwards of US$650,000 [30]. Additionally, NoV outbreaks in military settings pose security risks. Development of a vaccine would reduce economic costs, aid military operations, and save lives; however, development of a NoV vaccine faces considerable obstacles given the strain heterogeneity, likely necessitating a multivalent formulation. While it is encouraging that multivalent vaccines appear to elicit robust responses against strains that are not included in the cocktail [24], the molecular mechanisms governing these responses must be defined for rational vaccine design. There are several other important considerations that would aid in design of such a vaccine:

1. Mapping and determining the relative contribution of each blockade epitope as a correlate of short or long-term protective immunity is key to successful vaccine design, especially against strains that evolve over time. Mapping conserved intra- or intergenotype epitopes may uncover more broadly acting therapeutic targets.
2. Additional human challenge studies with both GI and GII NoVs need to be conducted to more fully understand how long protective immunity lasts among different genogroups and genotypes.
3. The impact of preexposure history and temporal and phylogenetic space on contemporary strain vaccine immune responses will need to be clarified.
4. The contribution of T cell responses (CD4+, CD8+, Th17) in protective immunity will need to be further elucidated.
5. The role of innate immunity in viral pathogenesis and in short- and long-term herd immunity will need to be examined. Areas of the capsid undergoing positive selection over time in rapidly evolving NoV genotypes will need to be continuously monitored in order to keep abreast of novel surface variation that may lead to escape from herd immunity and emergence of new pandemic strains.
6. The potential effects a vaccine would have on the evolutionary dynamics of emerging NoV strains will need to be clarified.

Addressing these questions will not only allow for better design of NoV vaccines and immunotherapeutics, but will inform strategies for minimizing the global disease burden of other highly variable and highly pathogenic human viruses.
